# Cytopathology whole slide images and adaptive tutorials for senior medical students: a randomized crossover trial

**DOI:** 10.1186/s13000-016-0452-z

**Published:** 2016-01-08

**Authors:** Simone L. Van Es, Rakesh K. Kumar, Wendy M. Pryor, Elizabeth L. Salisbury, Gary M. Velan

**Affiliations:** Department of Pathology, School of Medical Sciences, The University of New South Wales, Sydney, NSW 2052 Australia; Royal College of Pathologists of Australasia, Surry Hills, 2010 Australia; Department of Anatomical Pathology, Prince of Wales Hospital, Randwick, 2031 Australia

**Keywords:** Cytopathology, Virtual microscopy adaptive tutorials, Digital microscopy, Virtual microscopy, Virtual slides, Whole slide images, WSI, Pathology education

## Abstract

**Background:**

Diagnostic cytopathology is an essential part of clinical decision-making. However, due to a combination of factors including curriculum reform and shortage of pathologists to teach introductory cytopathology, this area of pathology receives little or no formal attention in most medical school curricula. We have previously described the successful use of efficient and effective digital learning resources, including whole slide images (WSI) and virtual microscopy adaptive tutorials (VMATs), to teach cytopathology to pathology specialist trainees – a group that had prior exposure to cytopathology in their day to day practice. Consequently, in the current study we attempted to demonstrate the efficiency and efficacy of this eLearning resource in a cohort of senior medical students that was completely naïve to the subject matter (cytopathology).

**Methods:**

We evaluated both the quantitative and qualitative impact of these digital educational materials for learning cytopathology compared with existing resources (e-textbooks and online atlases). The senior medical students were recruited from The University of New South Wales Australia for a randomized cross-over trial. Online assessments, administered after each arm of the trial, contained questions which related directly to a whole slide image. Two categories of questions in the assessments (focusing on either diagnosis or identification of cellular features) were utilized to determine efficacy. User experience and perceptions of efficiency were evaluated using online questionnaires containing Likert scale items and open-ended questions.

**Results:**

For this cohort of senior medical students, virtual microscopy adaptive tutorials (VMATs) proved to be at least as effective as existing digital resources for learning cytopathology. Importantly, virtual microscopy adaptive tutorials had superior efficacy in facilitating accurate diagnosis on whole slide images. Student perceptions of VMATs were positive, particularly regarding the immediate feedback, interactivity and equity of learning which this learning resource provides.

**Conclusions:**

Virtual microscopy adaptive tutorials have the potential to improve the efficacy of learning microscopic pathology for medical students. The enhanced learning experience provided by these eLearning tools merits further investigation of their utility for other cohorts, including specialist trainees.

## Background

Beginning with imprint smears in the 1830s and progressing to needle aspiration in the 1920s, diagnostic cytopathology has now become an essential part of clinical decision-making, with application to samples ranging from body fluids to solid tumour masses [1, 2]. Cytological examination is the basis for initial diagnosis of most tumors and many infections. It is also widely relied upon as an effective and relatively non-invasive screening tool for many diseases. Despite its importance, this is an area that receives little formal attention in medical school curricula, or, if it does, may be taught by non-pathologists [3, 4]. While most medical students will become practicing clinicians rather than pathologists, they will rely on diagnostic reports written in the language of cytopathology. Hence medical students need to acquire some basic concepts about how cytopathological diagnoses are rendered.

Whole slide images (WSI) have proved to be a reliable method for teaching both histology and histopathology [5–13] and are useful for diagnostic histopathology and cytopathology [14–16]. There are potential limitations for digital cytopathology, because WSI have difficulty displaying the z-axis. However, comparative studies have found no evidence of major inaccuracies [13, 14, 17–20]. Therefore, for educational purposes, WSI and associated technology are likely to suffice for cytopathology, even without z-axis capability. There is thus an unprecedented opportunity to provide medical students with a meaningful introduction to cytopathology using WSI and associated technology, but to date, development of such educational resources has not been reported. Our experience has shown that that focused exposure of medical students to quality pathology teaching (including electronic educational material) has a positive impact on their understanding of pathology, not only in terms of its application in clinical practice, but with respect to influencing their choice of pathology as a career pathway [21].

Virtual microscopy using WSI provides students with convenient access to consistent, high quality educational materials, and can be combined with so-called ‘intelligent tutoring systems’. We have previously reported the development of virtual microscopy adaptive tutorials (VMATs), which are interactive online tutorials for histology and cytology [13, 22]. These can promote learning by interaction and exploration of WSI. We have demonstrated that VMATs are beneficial both in terms of efficiency and the potential for personalized learning, and that students find these educational resources relevant, engaging and meaningful [22].

Recently, we evaluated the use of WSI without z-stacks and VMATs, based on these WSI, for education of specialist pathology trainees in Australasia [13]. We chose cytopathology as the subdiscipline to work with this technology for several reasons: high quality, standardized and equitable cytopathology teaching material is needed for teaching and examining cytopathology in Australasia and cytopathology specimens are challenging to digitize due the intrinsic 3D nature of the original specimen. Both important reasons to provide validation of a potentially effective and valuable teaching method for this subject matter. For specialist pathology trainees, VMATs were equally effective in teaching cytopathology compared to traditional methods of learning with glass slides, microscopes and textbooks. In addition, VMATS were perceived positively in comparison to traditional methods of learning cytopathology.

However, the cohort of pathology trainees, in the study described, already had exposure to some cytopathology training. It was difficult to control for this prior exposure in the specialist trainee cohort. Consequently, in the current study, we extended this approach to senior medical students who are cytopathology naïve, with the aim of evaluating the effectiveness and efficiency of the VMATs in teaching. We conducted a randomized cross-over trial in a cohort of senior medical students, to compare learning cytopathology with WSI and VMATs to learning from existing resources such as online textbooks, atlases and websites.

## Methods

### Whole slide images

These were acquired at × 40 magnification using an Aperio Scanscope XT (Leica Biosystems Nussloch GmbH). WSI for this trial were identical to those created for a similar trial with anatomical pathology trainee participants. Methodology for acquisition of these WSI has been previously described [13]. WSI were stored in a biomedical image database called Slice (https://www.best.edu.au/slice/featured). An example of the Slice interface can be accessed via: https://www.best.edu.au/s/ruj9leul/vadul81r.

### Development of VMATs

**Fig. 1:**
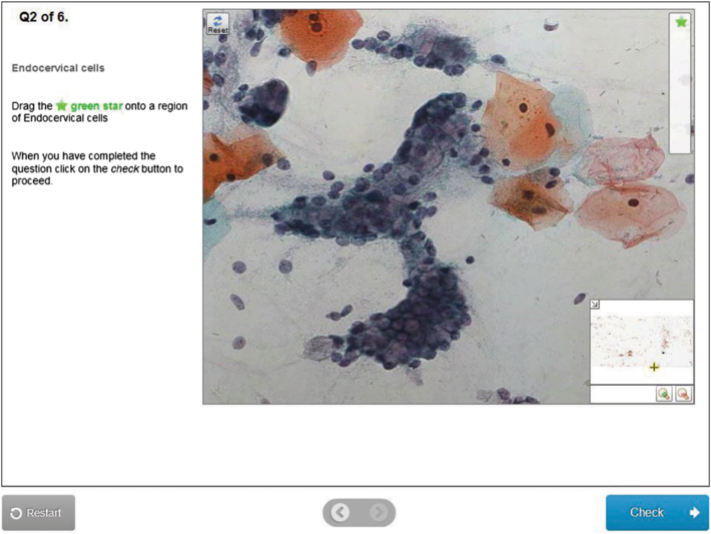
Example of an interactive question within a cytopathology VMAT. Abbreviations: VMAT, virtual microscopy adaptive tutorial

**Fig. 2:**
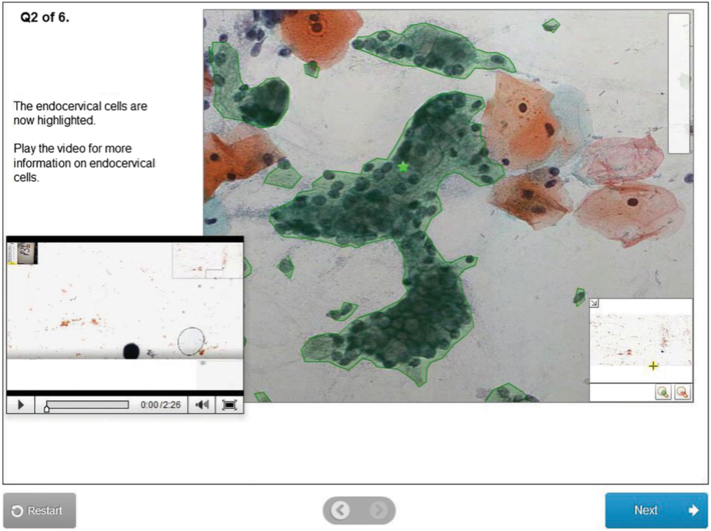
Example of immediate feedback within a cytopathology VMAT. Abbreviations: VMAT, virtual microscopy adaptive tutorial

VMATs can be created using the Adaptive e-Learning Platform (AeLP), an intelligent tutoring system developed by Smart Sparrow™ (https://www.smartsparrow.com/). This intelligent tutoring system has been previously described [13, 22–24]. We utilized 22 previously described VMATs [13] for the current trial. The VMAT interface can be seen in Figs. 1 and 2. An example VMAT can be accessed via the following link: https://aelp.smartsparrow.com/bronte/viewer/open/s581vrzn.

### Trial design and analysis

The trial was approved by the UNSW Human Research Ethics Committee (HC 14354). It focused on the same three main themes of cytopathology as in our previous trial with anatomical pathology trainees [13]. These included gynecology, fine needle aspiration (FNA) and exfoliative/effusion fluid cytopathology. The diagnostic categories of the 22 VMAT-supported cases have been previously listed [13]. Participants were volunteer year 5 and year 6 medical students from the 6-year undergraduate Medicine program at the University of New South Wales (UNSW) Australia, recruited by broadcast email.

Informed consent was obtained from all participants, who had the option of withdrawing at any time. No inducement was offered to the students to participate in the trial. Similar to the trial performed amongst anatomical pathology trainees [13], this trial was also divided into three phases as seen in Table 1, with a cross-over between phases and an online assessment at the end of each phase. The cross-over design was employed to account for any potential differences between groups with respect to knowledge, possible experience with cytology, and pre-trial familiarity with WSI. Diagnostic categories in the three online assessments (gynecological cytology, fine needle aspiration cytology, fluid/exfoliative cytology) and the assessment cases were identical to those described for the previous trial with anatomical pathology trainees [13]. Participants were randomized into two groups such that there were equal numbers of year 5 and year 6 students in each group. At the commencement of each one-week phase of the trial, each group either received links to WSI and VMATs, or to a list of diagnoses/disorders to be studied according to existing methods, including links to a comprehensive cytopathology e-textbook and online atlas via the UNSW library and to a well-known cytopathology educational website. Participants were asked to record the amount of time they spent studying cytopathology during each phase. In online questionnaires, participants were also asked to rate the overall value of WSI and VMATs as educational tools (on a scale from1 to 10) and to evaluate other aspects of their experience (using 5 point Likert scale items). The questionnaire also invited open-ended responses. These questionnaires were provided to all participants at the end of the first phase, to group 1 (intervention group) at the end of the second phase and to group 2 (intervention group) at the end of the third phase of the trial.

**Table 1:** Timeline and format of digital cytopathology trial for UNSW Medicine students (year 5 and 6)

Topic	Group 1	Group 2	Timeline (Days)
Gynecological cytology	VMATs and WSI	VMATs and WSI	0-7
	ONLINE ASSESSMENT – Gynecological cytology		8-11
Fine needle aspirate cytology	VMATs and WSI	Traditional	12-18
	ONLINE ASSESSMENT - Fine needle aspirate cytology		19-23
Fluid/Exfoliative cytology	Traditional	VMATs and WSI	24-30
	ONLINE ASSESSMENT – Fluid cytology		31-34

*Abbreviations*: *WSI* whole slide images, *VMATs* virtual microscopy adaptive tutorials, *UNSW* The University of New South Wales

Knowledge of relevant aspects of cytopathology that had been studied during that phase was assessed at the end of each phase by using a time-limited (60-min) online virtual cytopathology quiz authored in using Questionmark Perception™ (Questionmark Computing Ltd, London, UK). All questions within each assessment contained a link to a whole slide image. The slide did not always contain the screener’s diagnostic locator marks. Each assessment question fell into one of two categories: 1. Select a favoured diagnosis from a list of alternatives (“Diagnosis”); or 2. Identify cellular features (“Identification”).

Each assessment tested the subject matter that had been studied for that phase of the trial. Only one attempt was permitted for each assessment. Participants completed an online form agreeing to an honor code, forbidding use of any outside aids or assistance (e.g. consultation with colleagues, access to textbooks or internet sources). Answers to assessment questions were automatically submitted once the time limit for the assessment had been reached. Following submission of their answers, participants received immediate automated feedback on their performance and were given an explanation regarding the correct answers.

### Analysis of trial data

Analysis of trial data was carried out in a similar fashion to our previous trial [13]: data shown are mean ± standard deviation (SD) unless otherwise stated. To compare prior academic performance, based on mean weighted average mark (WAM), of the students in each group, Student’s t-test was used. Total scores in each assessment, scores on items in the “Diagnosis” and “Identification” categories within each assessment, as well as self-reported hours of study in each phase of the trial were compared in the same way. Median ratings for questionnaire items were compared using Mann–Whitney tests.

Common themes were flagged and independently identified by two authors (SLVE, GMV) in the written responses to the open-ended questionnaire items. These responses had been exported into a spreadsheet to facilitate the thematic analysis.

## Results

### Characteristics of the cohort

Initially, 46 senior medical students volunteered for the trial and 23 were randomized to each of Groups 1 and 2. However, possibly because cytopathology was non-assessable supplementary material, several students withdrew early. As a result, the number of participants who completed one or more assessments was reduced to 17 students in Group 1 and 18 students in Group 2. There was no significant difference between the two groups in terms of seniority, with 10 students from year 5 and 7 from year 6 students in Group 1, compared with 11 from year 5 and 7 from year 6 in Group 2. Prior academic performance, as indicated by mean weighted average mark, was indistinguishable between groups (*P* = 0.96). There were also no significant differences between groups with respect to mean time spent studying the material in each phase of the trial (For Phase 1, Group1 = 2.6 ± 1.3, *n* = 17, Group 2 = 2. 8 ± 1.1, *n* = 18; P = 0.5; For Phase 2 and 3,existing methods of study = 2.2 ± 1.4 h, *n* = 29; WSI/VMATs = 2.0 ± 1.2 h, *n* = 31; *P* = 0.3.

### Assessment

**Fig. 3:**
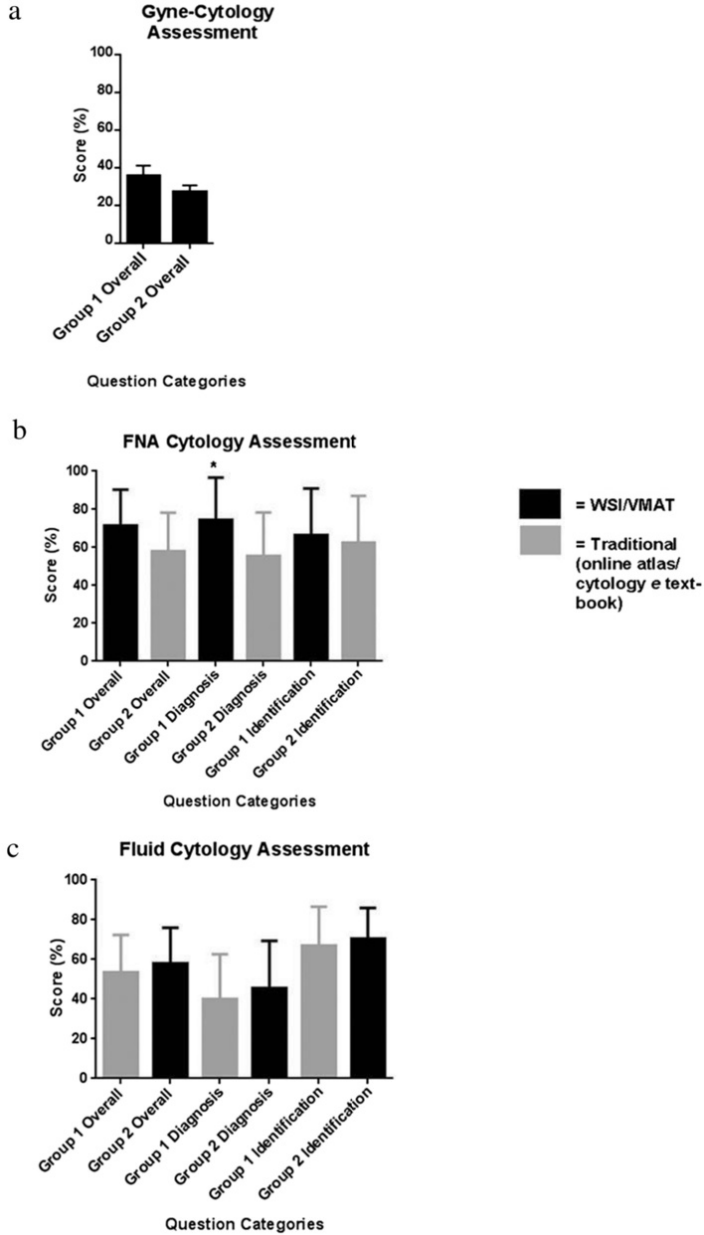
**a**,**b**,**c**: Group 1 and Group 2 assessment scores “Overall”, for “Diagnosis” questions and for “Identification” questions respectively, after studying for: (**a**) Gynecological cytology (**b**) FNA cytology and (**c**) Fluid/effusion cytology. (Please note: Gynecological cytology assessment consisted of “diagnostic” questions only; For FNA assessment, Group 1 = intervention group; For Fluid/effusion assessment, Group 2 = intervention group). Abbreviations: Gyne, Gynaecological; FNA, fine needle aspiration; WSI, whole slide image; VMAT, virtual microscopy adaptive tutorial; * = statistically significant result (*P* = 0.02)

In phase 1 of the trial, both groups were provided with the WSI and VMATs relating to gynecological cytopathology. There was no significant difference between groups in mean assessment scores (all questions for this assessment were in the “Diagnosis” category (Group 1 = 35.4 ± .24.3 %, *n* = 17; Group 2 = 27.1 ± 15.5 %, *n* = 18; *P* = 0.24) (Fig. 3a).

In phase 2 of the trial, which focused on FNA cytopathology, only Group1 received the WSI/VMAT resources, whilst Group 2 studied with existing resources. The mean assessment score of the intervention group was higher than the existing methods group, however this was not statistically significant (Group 1 = 71.7 ± 18.73 % *n* = 15; Group 2 = 58.2 ± 20.2, *n* = 18; *P* = 0.06). Further analysis revealed there was a significant difference in favor of Group1 for items in the “Diagnosis” category (Group 1 = 74.3 ± 22.4 %, *n* = 15; Group 2 = 55.6 ± 22.92 %, *n* = 18; *P* = 0.025), but not in the “Identification” category (Group 1 = 66. 7 ± 24.4 %, *n* = 15; Group 2 = 62.5 ± 24.6 %, *n* = 18; *P* = 0.63) (Fig. 3b).

In phase 3 of the trial, emphasizing fluid cytopathology, only Group 2 received the WSI/VMAT resources, whilst Group 1 studied with existing methods. There was no significant difference between groups in total assessment scores, (Group1 = 53.6 ± 18.8 %, *n* = 11; Group 2 = 58.0 ± 18.0 %, *n* = 16; *P* = 0.55). There were also no differences in mean scores for items in the “Diagnosis” category (Group 1 = 39.8 ± 22.9 %, *n* = 11; Group 2 = 45.3 ± 24.1 %, n = 16; *P* = 0.56) or the “Identification” category (Group 1 = 67.1 ± 19.6 %, *n* = 11; Group 2 = 70.30 ± 15.7 %, *n* = 16; *P* = 0.64) (Fig. 3c).

### Questionnaire ratings

**Fig. 4:**
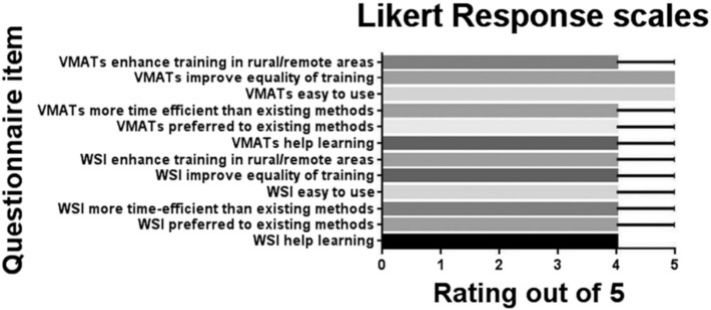
Perceived benefit of WSI and VMATs as a cytopathology learning tool expressed as median and range, where 5 = strongly agree and 1 = strongly disagree. Abbreviations: WSI, whole slide images; VMATs, virtual microscopy adaptive tutorials

While both WSI and VMATs were positively received, there was a significant preference for the VMATs (median rating 8 out of 10, *n* = 53) over WSI (7 out of 10, *n* = 58) (*P* < 0.0002). In Likert scale items (Fig. 4), VMATs were also perceived as more useful in developing diagnostic skills in cytopathology (*P* < 0.01), more time efficient (*P* = 0.01), and providing more equitable learning opportunities (*P* < 0.01) than WSI alone. There was a strong preference for both VMATs and WSI over existing methods of learning cytopathology, with a further significant preference for VMATs over WSI alone (*P* = 0.03).

### Open-ended questionnaire responses

Qualitative analysis of open-ended questionnaire responses resulted in the emergence of a number of themes. There were 84 comments on the VMATs (Table 2) and 101 comments on WSI during the three phases of the trial (Table 3). The dominant positive themes regarding VMATs were their value as a high-impact learning tool, benefits regarding equity of learning opportunities for students at rural campuses, immediate feedback and interactivity. One negative theme that emerged was the need for VMATs to be tailored to the level of understanding and training of the learner.

**Table 2:** Selection of stronger emerging themes for VMATs^a^

Themes	Response rate: N (%)
Valuable or High impact learning	41 (24)
Needs to be tailored to level of knowledge/training	25 (15)
Immediate feedback	18 (11)
Interaction	18 (11)
Equity of training	12 (7)
Convenient/Easy access (at home, in-transit)	11 (6.5)
Time efficient	10 (6)

^a^Comments containing multiple themes were given by many participants

*Abbreviations*: *VMATs* virtual microscopy adaptive tutorials

**Table 3:** Selection of stronger emerging themes for WSI^a^

Themes	Response rate: N (%)
Not enough to learn from in isolation – need VMATs or other	22 (13)
Convenient (at home, in-transit, on IPAD)	22 (13)
Intuitive interface /easy to use	17 (10)
Slides need annotations or “on-off” labels	15 (9)
Preference for screener’s marks	15 (9)
Better/easier than glass slides alone	14 (8.3)
Valuable or high impact learning	13 (7.7)
Equity of training	12 (7)
Resolution is good	10 (6)
Incorporate function to practice locator skills on slide and approach to a slide	9 (5)

^a^Comments containing multiple themes were given by many participants

*Abbreviations*: *WSI* whole slide images

With respect to WSI, positive themes related to convenience, ease of use, equity of learning opportunities and adequate visualization. Negative themes included the lack of interactivity and annotations, without which WSI were perceived as insufficient to learn cytopathology. None of the participants commented on the lack of z-axis capability.

## Discussion

The key findings to emerge from this study are firstly that by utilizing WSI and interactive learning tools such as VMATs, it is possible to provide medical students with a meaningful introduction to cytopathology, and it is possible to effectively engage students, even when the material being studied is difficult and supplementary to their curriculum. Secondly, the demonstration of the effectiveness of this method of learning, in a subject matter that is both difficult and completely new to the participants, provides strong support for the VMATs as an educational tool.

An interesting aspect of this trial was that we successfully introduced medical students to the diagnostic process employed by anatomical pathologists with respect to cytopathology samples. Learning about diagnostic cytopathology may help improve attention to key diagnostic features and enhance cognitive integration of multiple clues [25, 26]. Thus, introducing medical students in Australia to this sub-discipline may be valuable not only because of its “real world” relevance but also because it might enhance their overall diagnostic and decision-making skills. VMATs, which engage the learner by interaction and immediate informative tailored feedback, help to enhance the skills whilst remediating errors.

For this cohort of Australian senior medical students, VMATs proved to be at least as effective as existing digital methods for learning cytopathology, in terms of performance in subsequent assessments. These results are similar to those observed in a similar trial with pathology specialist trainees. Importantly however, in this trial, VMATs were superior in some respects in this cohort of senior medical students, e.g. ability to make an accurate diagnosis on a whole slide image. This superiority was clearly evident in the second phase of our trial, but was no longer apparent in the third phase, possibly because of a carry-over effect. A question that remains unanswered is whether students who learn interactively retain the material better, even if there was no immediate significant differences in assessment when comparing WSI/VMATs to existing learning techniques for cytopathology. Therefore, a follow up study after an adequate washout period would be helpful.

Similar to our findings in a similar trial with pathology specialist trainees, the student perceptions of VMATs were overwhelmingly positive. Key points to emerge from the analysis of questionnaires were that students regarded the best features of VMATs as their immediate feedback and interactivity. Without such interactions, WSI did not hold the same learning value for the current student cohort. In contrast, pathology trainees in our previous trial, emphasized how valuable the VMATs were as a learning tool in themselves and that WSI provided the flexibility and equity considered necessary by this specialist group for learning support [13]. There was also strong agreement amongst medical students that VMATs helped improve equity of learning between multiple clinical campuses, including rural campuses – also a strong theme amongst specialist trainees in our previous trial [13]. Both the pathology trainees in our previous trial and the medical students in the current trial rated WSI as highly convenient learning tools. While both WSI and VMATs were preferred to existing methods for learning, there was a significant preference for VMATs over WSI alone in this trial. Mean assessment scores were lower for the medical student cohort in the current trial in comparison to the corresponding mean assessment scores for the pathology trainees in our previous trial, supporting the notion that the medical student cohort were cytopathology-naïve.

We note that as these VMATS were originally authored for first and second year anatomical pathology trainees [13], it was unsurprising that some medical students felt they needed more introduction on how to approach a slide, a skill set taken for granted with pathology trainees. Nevertheless, the majority still considered the VMATs both straightforward to learn from and enjoyable. In contrast to other studies, including our own previous trial with postgraduate pathology trainees [13, 14, 18, 27], the lack of 3-D focus was not mentioned as an issue by this medical student cohort; although this is not surprising given this cohort have had no prior exposure to glass slides and microscopes.

Common to both this trial and the previous trial performed with anatomical pathology specialist trainees, participant performance improved with each successive assessment, suggesting that performance might have been influenced by increasing familiarity with the digital pathology environment. Such learning curves have been previously observed [16, 28].

We acknowledge several limitations of this study. Because of the optional nature of the material, the number of students who completed the assessments was modest. Consequently, this study may have been underpowered with respect to detecting an overall significant difference in performance between intervention and control groups in phase 2 and phase 3 of the trial. Other limitations include the fact that students were all from a single medical school and there was the also the potential impact of a selection bias - all participants were volunteers, which may reflect an interest in and positive perception of online learning technology such as WSI and VMATs.

## Conclusion

There are a number of implications from the findings of this study. Medical student teaching of microscopic pathology increasingly relies upon digital learning resources, and students now have high expectations of the quality of those resources. In this context, our findings may have broader implications. Using VMATs could open up opportunities to expand the breadth of teaching in microscopic pathology, including the complex area of cytopathology, even in a setting of limited face-to-face teaching time. Effective teaching and training such as encountered with the cytopathology VMATs may take the student one step further by showing them the complex process of integrating diagnostic features into a formulated diagnosis. Providing medical students with opportunities to understand the diagnostic process employed by anatomical pathologists in clinical practice has the potential to improve clinico-pathological correlation skills and thus ability to appropriately communicate with other health care providers on the patient’s results – a desirable skill for any doctor. The exposure to digital microscopy and such effective educational tools in pathology may also positively influence a student’s choice of pathology as a career pathway [21]. Digital educational tools in pathology that have been demonstrated to be effective in this and previous studies by our group [13, 22] may help provide the training in digital pathology which is becoming a requisite for medical graduates [29, 30]. Lastly, the demonstration of the effectiveness of this method of learning in a subject matter that is both complex and completely new to the participants in the study is strong support for the VMATs as an educational tool, the use of which could be trialed to improve the efficacy and efficiency of learning for specialist trainees in Pathology.

### Ethics

Prior to the commencement of this project, Ethics approval had been obtained (UNSW HC 14354) and approval received from Royal College of Pathologists of Australasia (RCPA).

## Abbreviations

WSI: Whole slide images
VMATs: Virtual microscopy adaptive tutorials
RCPA: The Royal College of Pathologists of Australasia
UNSW: The University of New South Wales
